# Insulin modulates the frequency of Ca^2+^ oscillations in mouse pancreatic islets

**DOI:** 10.1371/journal.pone.0183569

**Published:** 2017-08-28

**Authors:** Boah Lee, Taegeun Song, Kayoung Lee, Jaeyoon Kim, Per-Olof Berggren, Sung Ho Ryu, Junghyo Jo

**Affiliations:** 1 Department of Life Sciences, Pohang University of Science and Technology, Pohang, Gyeongbuk, Korea; 2 Asia Pacific Center for Theoretical Physics, Pohang, Gyeongbuk, Korea; 3 School of Interdisciplinary Bioscience and Bioengineering, Pohang University of Science and Technology, Pohang, Gyeongbuk, Korea; 4 The Rolf Luft Research Center for Diabetes and Endocrinology, Karolinska Institute, Stockholm, Sweden; 5 Department of Physics, Pohang University of Science and Technology, Pohang, Gyeongbuk, Korea; Universidad Miguel Hernandez de Elche, SPAIN

## Abstract

Pancreatic islets can adapt to oscillatory glucose to produce synchronous insulin pulses. Can islets adapt to other oscillatory stimuli, specifically insulin? To answer this question, we stimulated islets with pulses of exogenous insulin and measured their Ca^2+^ oscillations. We observed that sufficiently high insulin (> 500 nM) with an optimal pulse period (~ 4 min) could make islets to produce synchronous Ca^2+^ oscillations. Glucose and insulin, which are key stimulatory factors of islets, modulate islet Ca^2+^ oscillations differently. Glucose increases the active-to-silent ratio of phases, whereas insulin increases the period of the oscillation. To examine the dual modulation, we adopted a phase oscillator model that incorporated the phase and frequency modulations. This mathematical model showed that out-of-phase oscillations of glucose and insulin were more effective at synchronizing islet Ca^2+^ oscillations than in-phase stimuli. This finding suggests that a phase shift in glucose and insulin oscillations can enhance inter-islet synchronization.

## Introduction

Plasma glucose and insulin concentrations oscillate with a period of 5–10 min [1], similar to many other biochemical oscillations and cellular rhythms [2]. Glucose stimulates pancreatic β cells to secrete insulin, whereas insulin stimulates peripheral tissues and the liver to decrease glucose levels by glucose uptake and glycogen synthesis from glucose, respectively. The negative feedback loop modulates the glucose and insulin oscillations. However, the slow oscillation originates from β cells, which spontaneously generate oscillations in intracellular Ca^2+^, the major stimulator of insulin secretion [3–5], even under constant glucose stimuli [6]. However, oscillatory glucose can modulate the intrinsic oscillation of β cells, and make their Ca^2+^ oscillations to follow the external glucose oscillation [7–10]. This phenomenon is called glucose entrainment, and proposed to be an important mechanism for the synchronous hormone secretion from different islets in the pancreas [7, 11, 12].

Islet cells can respond to multiple nutrient, hormonal and neuronal signals [13], perhaps to integrate the external/internal information and regulate their internal energy homeostasis. Neighboring islet cells locally communicate through autocrine/paracrine interactions [14]. Insulin is one of the representative autocrine factors as insulin receptors are expressed on β cells [13]. It is an interesting autocrine factor that affects its own secretion, although whether the autocrine interaction is positive or negative is still debated [13, 15, 16]. No effect of insulin on insulin secretion has also been reported [17].

If islets can respond to insulin, especially oscillatory insulin, then our first question is whether the oscillatory insulin can entrain islets to produce synchronous hormone pulses in a similar manner as oscillatory glucose does [10]. Second, if glucose and insulin oscillations can entrain islets to follow their rhythms, we ask how the phase relation between the two oscillations affects the entrainment of the islets. We address the first question by performing Ca^2+^ imaging experiments and explore the second question by developing a mathematical model based on the experimental observations.

## Results

### Insulin modulates islet Ca^2+^ oscillations

**Fig 1:**
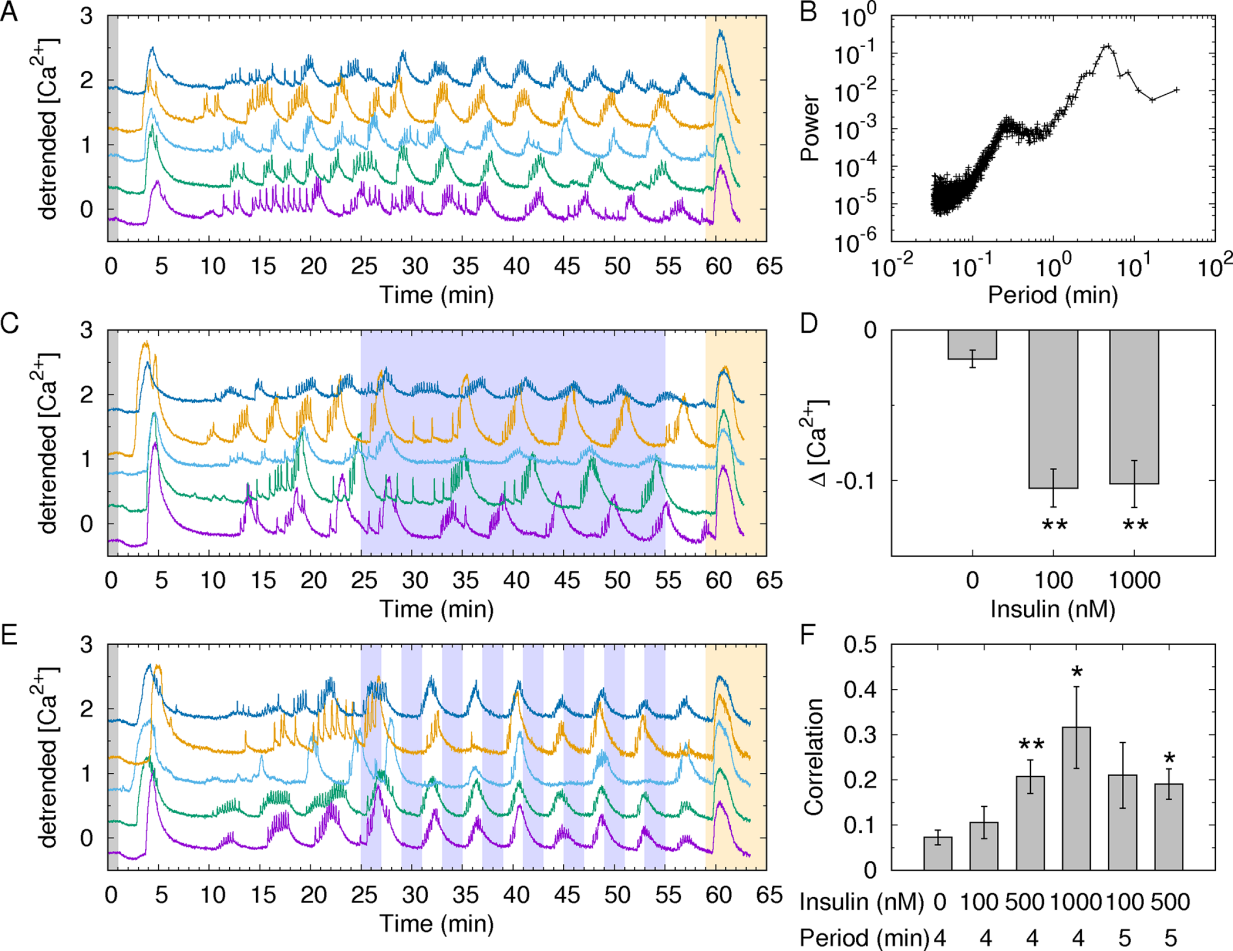
Modulated Ca^2+^ oscillations by insulin stimuli. (A) Time traces of the intracellular Ca^2+^ concentrations (340/380 nm fluorescence ratio) within each islet at 8.3 mM glucose stimulated from the basal concentration of 3 mM (gray region) at 1 min. To check the vitality of the islets at the end, the islets were depolarized with 25 mM KCl (orange region) at 59 min. The Ca^2+^ traces were normalized to have a zero mean value, and their linear trend was removed. Note that the Ca^2+^ traces were shifted except for the purple trace to avoid overlaps. (B) Power spectrum of the Ca^2+^ oscillations at 8.3 mM glucose in the absence of exogenous insulin stimuli. The two peaks represent fast (~ 30 sec) and slow (~ 4–5 min) Ca^2+^ oscillations. The largest peak was identified to define the dominant period of each Ca^2+^ oscillation. (C) From 25 min to 55 min, 1000 nM insulin was constantly infused (blue region). (D) Average activity change Δ[Ca^2+^] was defined as the difference of average [Ca^2+^] activities before (20–25 min) and after (30–35 min) insulin infusion. For the analysis, 68 (*n* = 6, *m* = 4), 62 (*n* = 5, *m* = 3), and 31 (*n* = 2, *m* = 2) Ca^2+^ traces were measured for 0, 100, and 1000 nM insulin, respectively, where *n* represents the number of Ca^2+^ imaging experiment and *m* represents the number of mice for the experiment. At each imaging, the Ca^2+^ profiles of 11±4 islets were simultaneously recorded. (E) For 2 min with a 4-min period, 1000 nM insulin was alternately infused. (F) The average correlations between every pair of Ca^2+^ traces per experiment were calculated to quantify the degree of synchronization between the Ca^2+^ oscillations for various protocols of insulin infusion. Two alternating periods were considered: 4 min (2 min with insulin and 2 min without insulin) and 5 min (2.5 min for each condition). The experiment was repeated *n* = 6, 5, 5, 5, 6, and 7 times (*m* = 3–4 mice) for each protocol (from left to right). Error bars represent standard errors. Student’s *t*-test was conducted to compare the first protocol (0 nM insulin and a 4-min period) with the corresponding protocols in (D) and (F). *P < 0.05. **P < 0.01.

To probe the response of islets to exogenous insulin, we measured their Ca^2+^ oscillations following exposure to 8.3 mM glucose. We chose this glucose concentration to minimize the contribution of endogenously secreted insulin while retaining stable Ca^2+^ oscillations. At 8.3 mM glucose, the islets generated mixed Ca^2+^ oscillations with fast spikes and slow oscillations, with a period of approximately 5 min (Fig 1A), a result similar to that reported in a previous study [18]. To quantify the characteristic periods of the Ca^2+^ oscillations, we used Fourier transform of the time traces, and obtained their power spectrum (Fig 1B). Then, we confirmed two peaks for the fast and slow components of the Ca^2+^ oscillations at 30 sec and 4–5 min, respectively. In this study, we focused on the slow component corresponding to the largest peak in the power spectrum.

When we stimulated the islets with a constant infusion of 1000 nM insulin, their Ca^2+^ oscillations were slightly suppressed at the initial insulin infusion but were not completely abolished (Fig 1C). We quantified the initial suppression by comparing the average Ca^2+^ activities for five minutes before (from 20 to 25 min) and (from 30 to 35 min) after the insulin infusion (Fig 1D). Here we excluded the time period (from 25 to 30 min) right after the infusion to consider the time delay for the insulin response. The average Ca^2+^ activities were inhibited under 100 and 1000 nM insulin. This result is consistent with the previous reports that those insulin concentrations inhibit insulin secretion [19, 20].

To prevent the desensitization of the insulin receptors [21, 22] and probe the rhythmic adaptation of the islets, now we stimulated the islets with alternating insulin infusions. When 1000 nM insulin was alternately infused for 2 min with a period of 4 min, the Ca^2+^ oscillations followed to the alternating stimuli and became synchronous between islets (Fig 1E). However, the inter-islet synchronization was not always so obvious. Therefore, we quantified the degree of synchronization between the Ca^2+^ oscillations by calculating their correlation. As a control experiment to exclude the effect of physical stimuli by solution exchanges, we exchanged the same glucose solution without exogenous insulin infusion with a period of 4 min, and confirmed that then the Ca^2+^ oscillations were not correlated. Furthermore, we checked that the mere solution exchange of constant 1000 nM insulin could not produce correlated Ca^2+^ oscillations of which correlation coefficient was 0.10±0.04. However, when the infused insulin concentration was alternated between 0 nM and 1000 nM, the Ca^2+^ oscillations became correlated. As the concentration of infused insulin increased from 100 to 1000 nM, the Ca^2+^ oscillations became more correlated in a dose-dependent manner (Fig 1F). This finding indicates that oscillatory insulin can make islets to produce synchronous Ca^2+^ oscillations. When the alternating period was changed from 4 min (2 min with insulin and 2 min without insulin) to 5 min (2.5 min for each), the inter-islet synchronization was still significant for a 500 nM insulin infusion but was not statistically significant for an 100 nM insulin infusion.

**Fig 2:**
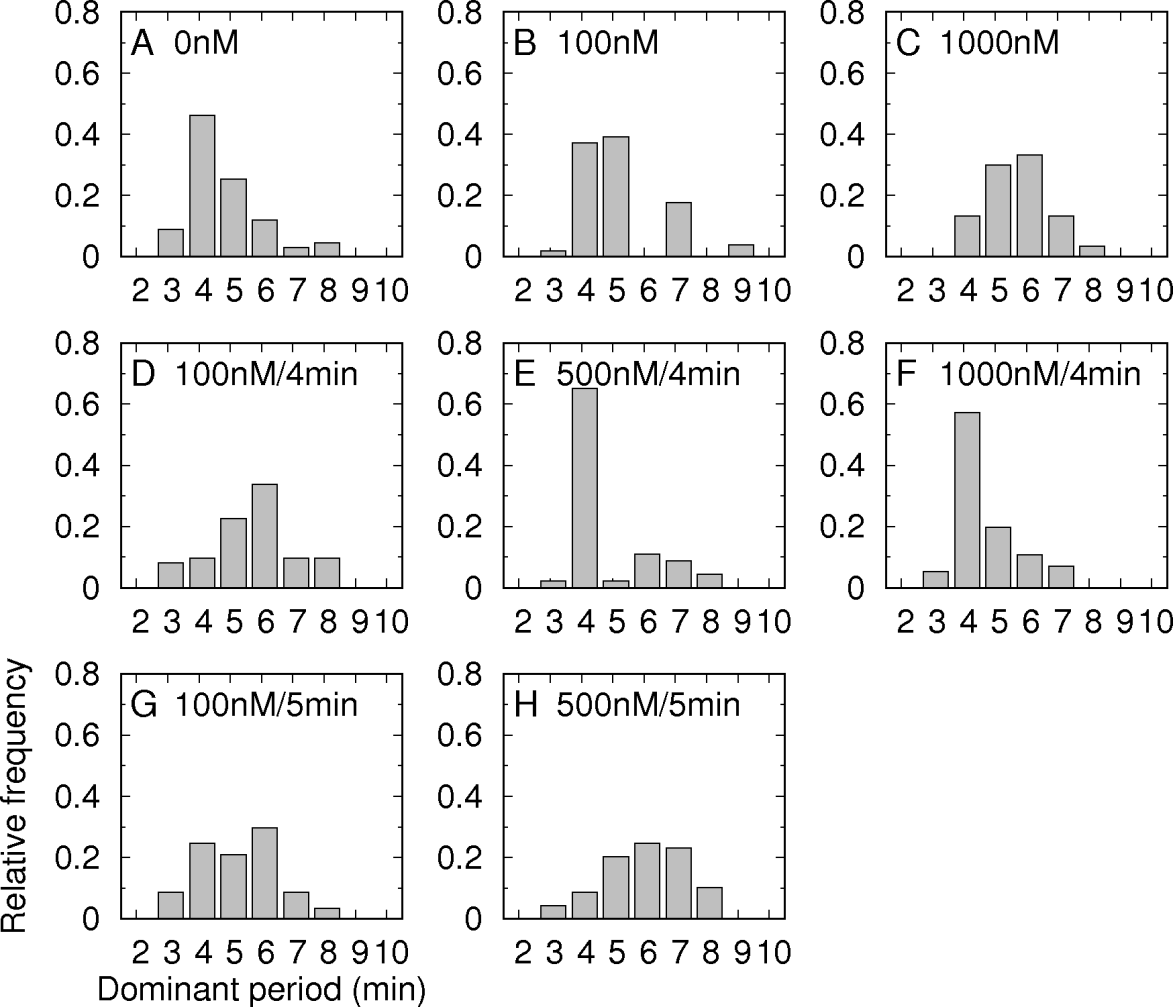
Dominant periods of Ca^2+^ oscillations under various insulin stimuli. The relative frequencies of the dominant Ca^2+^ periods for each protocol were plotted for the constant infusion of (A) 0 nM (68 islets), (B) 100 nM (62 islets), and (C) 1000 nM (31 islets) insulin; for the alternating infusion of (D) 100 nM (66 islets), (E) 500 nM (47 islets), and (F) 1000 nM (57 islets) insulin with a 4-min period (2 min with insulin and 2 min without insulin); and for the alternating infusion of (G) 100 nM (60 islets) and 500 nM (71 islets) with a 5-min period (2.5 min with insulin and 2.5 min without insulin).

To further validate the observed modulation by insulin, we examined whether the Ca^2+^ oscillations followed the alternating period of exogenous insulin. We identified the dominant period of the Ca^2+^ oscillations that had the largest peak in the power spectrum (Fig 1B). In the absence of exogenous insulin (Fig 1A), the dominant period of the Ca^2+^ oscillations was 4.6±1.1 min (Fig 2A). However, under constant infusion of 100 nM insulin, the dominant period was delayed to 5.3±1.2 min (Fig 2B). When the insulin concentration was further increased to 1000 nM, the Ca^2+^ oscillations were slow down (Fig 1C), and their corresponding dominant period was delayed to 5.8±1.8 min (Fig 2C). The delayed periods at 100 and 1000 nM insulin were significantly longer than the period in the absence of insulin (P<0.01). When we infused 100 nM insulin alternately with a 4-min period, the dominant periods shifted to longer periods than the periods under no insulin infusion (Fig 2D). However, when the concentration of infused insulin was further increased to 500 and 1000 nM, the dominant periods of the Ca^2+^ oscillations became mostly 4 min and followed the alternating period of insulin infusion (Fig 2E and 2F). This finding again demonstrated that sufficiently high insulin could entrain islets to follow its alternating rhythm. Next when we infused 100 and 500 nM insulin with a slightly longer period of 5 min, the dominant periods were broadly distributed at approximately 5–6 min (Fig 2G and 2H). This finding is distinct from the sharp distribution of the dominant periods for the 4-min alternation of insulin infusion, which suggests that the 4-min period might be optimal for the entrainment. Glucose entrainment has been shown to exhibit an optimal alternating period, which is neither too fast nor too slow [8, 10, 23, 24].

### Phase shift of glucose and insulin oscillations enhances inter-islet synchronization

**Fig 3:**
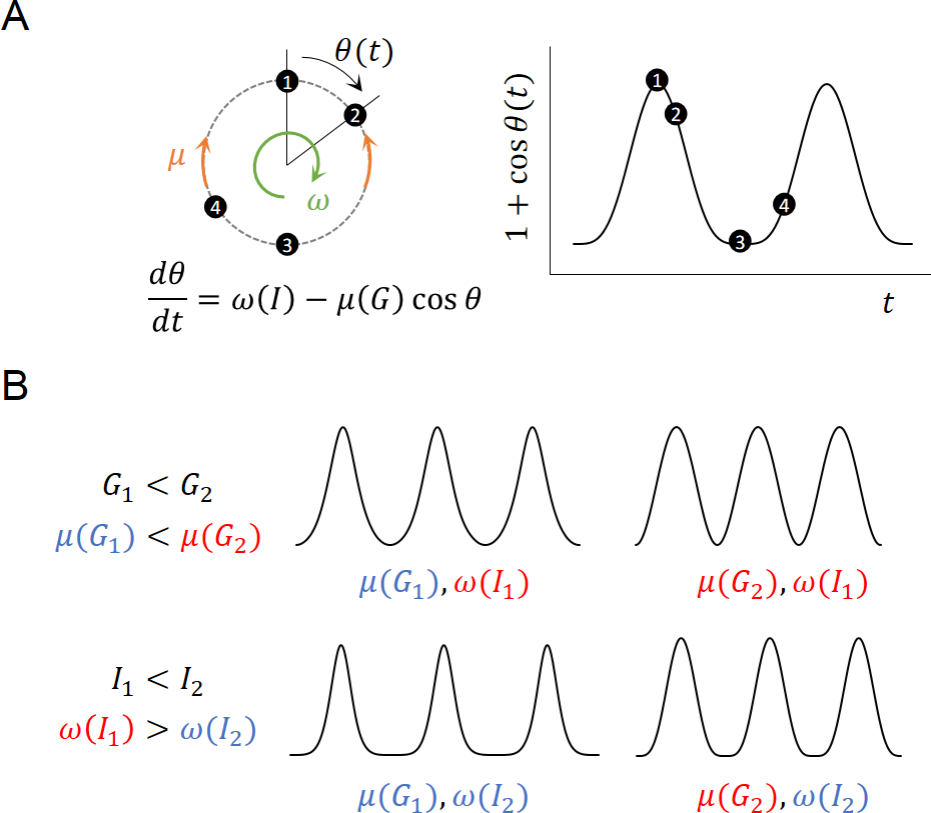
Active rotator model and the modulation of Ca^2+^ oscillations by glucose and insulin. (A) An angular movement with a phase θ(*t*) of a rotator can describe the temporal profile of the oscillations: 1 + cosθ(*t*) with time *t*, where the unity was introduced to prevent negative value during the Ca^2+^ oscillations. Glucose modulates mainly the active-to-silent ratio of phases through the pinning force μ(*G*), whereas insulin modulates the period of the oscillations through the intrinsic angular velocity *ω*(*I*). (B) Given a fixed insulin level, high glucose modulates the rotator to stay longer in active phases by increasing *μ*(*G*). By contrast, given a fixed glucose level, high insulin modulates the rotator to stay longer in silent phases by decreasing *ω*(*I*). Therefore, the combination of high (low) glucose and low (high) insulin can maximally modulate the rotator to stay longer in active (silent) phases. However, the in-phase combination of glucose and insulin is incompatible to drive the rotator to the same phase.

Prior studies have shown that glucose can modulate the plateau fraction (ratio of active-to-silent phases) of islet Ca^2+^ oscillations but their periods are minimally perturbed [10, 25, 26]. By contrast, in our study, insulin significantly modulated the period of islet Ca^2+^ oscillations, whereas insulin minimally perturbed the plateau fraction. The constant infusion of 1000 nM insulin increased the oscillation period from 4.6±1.1 min to 5.8±1.8 min (P<0.001). However, the plateau fraction slightly decreased from 0.39±0.03 to 0.36±0.04 (P<0.001). These observations can be mathematically described using the following active rotator model [27]:

$$\frac{d\theta }{dt}=\omega (I)-\mu (G)\cos\theta ,$$

which describes the time evolution of the rotator phase θ(*t*). The phase velocity ω(*I*) mainly controls the oscillation period, whereas the pinning force μ(*G*) mainly modulates the plateau fraction of the oscillation. Therefore, the time evolution of the rotator phase θ(*t*) can describe the Ca^2+^ oscillation: 1 + cosθ(*t*), where unity is added to prevent a negative Ca^2+^ concentration (Fig 3A). Here, the phases θ = 0 and θ = π correspond to the peak and valley of the Ca^2+^ oscillation, respectively. To incorporate the experimental observations of the frequency and phase modulations by insulin and glucose, we defined ω(*I*) as a decreasing function of the insulin concentration *I*, and μ(*G*) as an increasing function of the glucose concentration *G* (Materials and Methods). Because high glucose increases the pinning force μ(*G*), the rotator remains at θ = 0 (active phase) for a longer duration. Next, because high insulin decreases the phase velocity ω(*I*), the rotator rotates slowly.

Thus, the simple rotator model captured the modulations of the Ca^2+^ oscillation by glucose and insulin. Equipped with this mathematical model, we explored the following question: How does the Ca^2+^ oscillation adapt to the dual modulation of glucose and insulin? We consider two situations in which pulsatile glucose and insulin stimuli are (i) in phase and (ii) out of phase. The rotator model can predict the modulation of the Ca^2+^ oscillation depending on the relative levels of glucose and insulin (Fig 3B). First, given a fixed insulin level, high glucose modulates the Ca^2+^ oscillation to stay longer in active phases by increasing the ratio *μ*(*G*_2_)/*ω*(*I*) > *μ*(*G*_1_)/*ω*(*I*) for *G*_2_ > *G*_1_. By contrast, given a fixed glucose level, high insulin modulates the Ca^2+^ oscillation to stay longer in silent phases by decreasing the ratio *μ*(*G*)/*ω*(*I*_2_) < *μ*(*G*)/*ω*(*I*_1_) for *I*_2_ > *I*_1_. Note that here we consider moderately high glucose conditions, *G* < 11.6 mM, where *μ*(*G*) < 0 is negative [10]. Therefore, low (high) glucose can create a synergy effect with high (low) insulin to drive the Ca^2+^ oscillation to silent (active) phases. By contrast, in-phase conditions (low glucose and low insulin, or high glucose and high insulin) are incompatible to drive the Ca^2+^ oscillation to the same phase. Therefore, the out-of-phase coordination of glucose and insulin pulses can be more effective for the Ca^2+^ oscillation to follow their rhythms.

**Fig 4:**
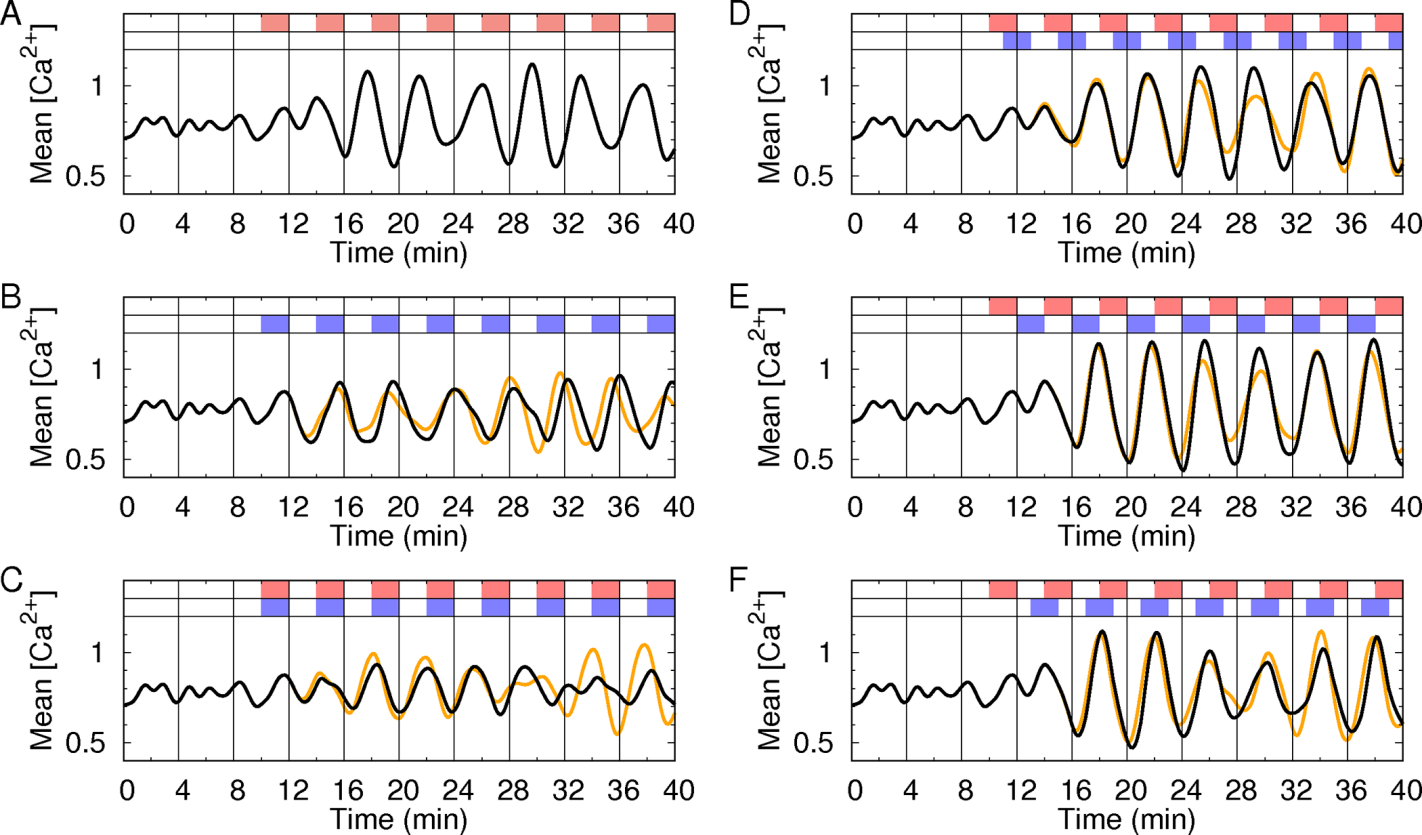
Synchronization of simulated Ca^2+^ oscillations under alternating glucose and insulin stimuli. (A) One hundred active rotators oscillated with heterogeneous intrinsic periods (4 to 5 min) starting from random initial phases. At 10 min, the basal glucose concentration *G*_1_ = 8.3 mM (white regions) was alternated with the *G*_2_ = 8.8 mM glucose concentration (red regions). To represent the degree of synchronization (or entrainment) between rotators, the mean value (black line) of the one hundred Ca^2+^ oscillations was plotted. When the synchronization between rotators was poor/good, the average value became flat/oscillatory. (B) Instead of the alternation of glucose, at 10 min, *I*_2_ = 1000 nM (black line) or *I*_2_ = 100 nM (orange line) was alternately infused with *I*_1_ = 0 nM for 2 min with a 4-min period (blue regions). Then, both glucose and insulin were alternately infused with phase differences of (C) 0, (D) *π*/2, (E) *π*, and (F) 3π/2 radians. Basal glucose (8.3 mM, white regions), stimulatory glucose (8.8 mM, red regions) and insulin (1000 or 100 nM, blue regions) were used (See Materials and Methods for the details of the simulations).

To test this hypothesis, we simulated the Ca^2+^ oscillation under a dual modulation of alternating *G*(*t*) and *I*(*t*). We set *G*(*t*) to alternate between *G*_1_ = 8.3 and *G*_2_ = 8.8 mM with a period of 4 min. The amplitude Δ*G* = *G*_1_−*G*_2_ = 0.5 mM is the minimal level necessary to entrain the Ca^2+^ oscillation [10]. Then we set *I*(*t*) to alternate between *I*_1_ = 0 and *I*_2_ = 1000 nM with the same period. Note that the values of *ω*(*I*_1_), *ω*(*I*_2_), *μ*(*G*_1_), and *μ*(*G*_2_) were experimentally determined (Materials and Methods). First, we confirmed that alternating *G*(*t*) or *I*(*t*) alone could entrain the Ca^2+^ oscillations to follow the alternation (Fig 4A and 4B). Second, as predicted, when *G*(*t*) and *I*(*t*) alternated in phase, the entrainment of the Ca^2+^ oscillations was diminished and became even worse than that with either *G*(*t*) or *I*(*t*) alone (Fig 4C). By contrast, when *G*(*t*) and *I*(*t*) alternated with phase shifts of *π*/2, *π*, or 3π/2, the entrainment was enhanced to show synchronous oscillations between rotators (Fig 4D–4F). Finally, we checked a lower concentration of insulin could still enhance the entrainment of the Ca^2+^ oscillations given alternating glucose. Then, we found that 100 nM insulin could enhance the entrainment, although the effect was slightly reduced.

## Discussion

In this study, we showed that an alternating stimulus of exogenous insulin could entrain the Ca^2+^ oscillations of islets to follow the alternation rhythm when the insulin concentration was sufficiently high (> 500 nM) and that the alternation period was close to the intrinsic period (~ 4 min) of the Ca^2+^ oscillations. Therefore, both glucose and insulin can modulate and entrain the Ca^2+^ oscillations. However, glucose mainly modulated the phase (or shape) of the Ca^2+^ oscillations, whereas insulin modulated the frequency (or period). Adopting the active rotator model, we mathematically described the phase and frequency modulation of glucose and insulin, and simulated the dual stimuli. Using this model, we found that a phase shift between the glucose and insulin oscillations could enhance the entrainment of the Ca^2+^ oscillations.

We have previously found that a simple mathematical model of oscillators could nicely capture the essential feature of the phase modulation of the islet Ca^2+^ oscillations by glucose [10]. In this study, we extended the active rotator model by incorporating the frequency modulation of the islet Ca^2+^ oscillations by insulin. Islet cells are information integrators that sense multiple nutrients, hormonal signals and neuronal signals for the robust regulation of glucose homeostasis. The rotator model could be generally applied to describe the Ca^2+^ oscillations modulated by other autocrine/paracrine signaling factors beyond glucose and insulin, if the modulation is about the phase and frequency of the Ca^2+^ oscillations. The slow Ca^2+^ oscillation originates from the glycolytic oscillation [18]. It would be interesting to investigate which molecular pathways the glucose/insulin signals perturb to modulate the phase/frequency of the metabolic oscillation. In particular, insulin has multiple pathways that can perturb cytosolic Ca^2+^ concentration. For instance, insulin increases the cytosolic Ca^2+^ level through nicotinic acid-adenine dinucleotide phosphate (NAADP)-sensitive Ca^2+^ stores, although the increased Ca^2+^ is not accompanied by insulin secretion [28]. In addition, insulin hyperpolarizes β cells by activating ATP-sensitive K^+^ channels, and abolishes the Ca^2+^ oscillations via phosphatidylinositol 3-kinase [19].

Here the autocrine effect of insulin on insulin secretion is controversial [13, 15, 16, 29]. Aspinwall et al. showed that 200 nM insulin leads to an increased intracellular Ca^2+^ concentration at 3 mM glucose [30, 31], whereas Khan et al. observed that 100 nM insulin inhibits the Ca^2+^ oscillations at 10 mM glucose [19]. However, Luciani et al. observed no perturbing effect of 200 nM insulin on the Ca^2+^ oscillations at 10 mM glucose [17]. Furthermore, Ueki et al. demonstrated the positive feedback of insulin; they observed diminished insulin secretion in mice with insulin and IGF-1 receptors specifically knocked out in β cells [32]. The insulin effect appears to be complex; it depends on the stimulating concentration of insulin [20], the environmental conditions, such as the glucose level, and the experimental protocols. Nevertheless, the expression of insulin receptors in β cells [13] strongly implies the potential for an autocrine interaction of insulin. In this study, we observed that insulin modulated the period of the Ca^2+^ oscillations, and the stimulus of alternating insulin made islets to generate synchronous Ca^2+^ oscillations. Our observation suggests that insulin stimuli can modulate the temporal profiles of insulin secretion in addition to causing a possible amplitude modulation.

Although we observed that 100 nM insulin could modulate the frequency of the Ca^2+^ oscillations, higher insulin concentration (> 500 nM) was required to entrain Ca^2+^ oscillations in addition to the frequency modulation. The effective concentration of exogenous insulin was relatively high (> 500 nM), compared with the physiological concentration, which is in the range of only a few nanomolars in blood circulation. Here we might need to consider that both glucose and insulin levels always fluctuate under in vivo conditions. Although a very high concentration of insulin was required to induce inter-islet synchronization purely by alternating insulin stimuli, a low concentration of insulin might be sufficient to enhance the inter-islet synchronization primarily induced by alternating glucose stimuli.

The synergy of the dual stimuli was maximal when oscillatory glucose was out of phase with oscillatory insulin. High glucose modulates the Ca^2+^ oscillations to stay longer in active phases, whereas high insulin slows down the Ca^2+^ oscillations. The delayed oscillation modulates the Ca^2+^ oscillations to stay longer in silent phases (Fig 3). Thus in-phase stimuli of glucose and insulin become incompatible to entrain the Ca^2+^ oscillations for staying at the same phase. By contrast, high (low) glucose and low (high) insulin stimuli become synergistic to modulates the Ca^2+^ oscillations for staying longer in active (silent) phases. Indeed, plasma glucose and insulin oscillate with a phase shift. This result could be a product of the negative feedback between the insulin secretion in response to glucose and the glucose regulation by insulin. Glucose oscillations occur ahead of the insulin oscillations, with a *π*/5 shift in monkeys [33] and with a *π*/3 shift in humans [1]. Although the phase shift can be an inevitable result of the glucose-insulin feedback, our model suggests that the phase shift can have the functional advantage of better entraining the Ca^2+^ oscillations, although the hypothesis remains to be tested by experiment.

In this study, we focused on the role of insulin for the temporal modulation of islet Ca^2+^ oscillations and inter-islet synchronization. However, the insulin signaling on insulin secretion can also affect intra-islet synchronization. The local insulin concentration in the extracellular space can be high. Braun et al. have estimated the local concentration to be in the micromolar range, and they hypothesized that the autocrine interaction between β cells within each islet could play a role in synchronizing their insulin secretion [15]. Our observation of the entrainable Ca^2+^ oscillations to insulin pulses supports this hypothesis of intra-islet synchronization. However, further study needs to elucidate the insulin and Ca^2+^ signaling on a molecular level for the frequency modulation of Ca^2+^ oscillations by insulin.

## Materials and methods

### Experimental animals and islet preparation

All of the experimental procedures were approved by the Pohang University of Science and Technology Institutional Animal Care and Use Committee (POSTECH IACUC, Korea). C57BL/6J male mice (Jackson Laboratory, Bar Harbor, ME, USA) aged 8–12 weeks were used. The animals were maintained under a 12 h light/dark cycle with free access to water. Mice were sacrificed by cervical dislocation under anesthesia with CO_2_. Islets were isolated from the pancreas of male C57BL/6J mice (25–30 g) after collagenase digestion. Briefly, 3 mL of 1 mg/mL collagenase P was injected into the pancreas via the common bile duct or by direct injection to expand the pancreas. The pancreas was excised and cut into small pieces, which were then digested with collagenase P to obtain free islets. With the aid of a dissecting microscope, islets were hand-picked and the islets were then placed in RPMI 1640 that contained 11 mM glucose, 10% fetal bovine serum, and 1% penicillin-streptomycin. Islets were cultured at 37°C in a 95% O_2_ and 5% CO_2_ mixture for one day before the experiment.

### Measurements of Ca^2+^ oscillations in islets

To monitor the intracellular concentrations of the Ca^2+^ ions, each batch of islets was incubated in a buffer solution that contained 3 mM glucose with 2 μM fura-2/AM at 37°C and 5% CO_2_ for 30 min. After incubation, the islets were attached to coverslips using Puramatrix Hydrogel (BD Biosciences, Bedford, MA). The islets were placed in an open perifusion chamber (Live cell instrument, Seoul, South Korea). The chamber was mounted onto an inverted fluorescence microscope (IX71, Olympus, Tokyo, Japan) and maintained at 37°C. All of the buffer solutions with various glucose/insulin concentrations were delivered to the chambers that contained the islets using a fast-flow solution-switching system (VC6; Warner Instruments, Hamden, CT). The flow rate of this system was 3 mL/min, and all of the solutions were maintained at 37°C. Intracellular Ca^2+^ was measured using the 340/380 nm fluorescence ratio with a fluorescence microscope. The microscope was equipped with a 300-W xenon arc lamp (Lambda DG-4, Sutter Instruments, Novato, CA) that contained the appropriate filters for the excitation of fura-2 at 340 nm and 380 nm. Fluorescence images were acquired with a 100 ms exposure every 1 sec by a CCD camera (ImagEM, Hamamatsu Photonics, Hamamatsu, Japan). The 340/380 nm fluorescence ratios for all islets were obtained and analyzed using MetaFluor software (MDS Analytical Technologies, Sunnyvale, CA). Each islet was defined as a region of interest (ROI). Signals from the ROI were corrected by subtracting the background signal. Islets were perfused within the chamber with 3 mM glucose for 5 min prior to recording the fura-2/AM fluorescence. We used two protocols to measure the islet Ca^2+^ oscillations under constant and alternating insulin conditions. The first protocol started with the glucose concentration at a basal level (3 mM), which was followed by an increase to a higher glucose concentration (8.3 mM) from 160 sec to 1600 sec. After the equilibration, a constant concentration (0, 100, or 1000 nM) of exogenous insulin was infused for 1800 sec. Note that the case of 100 nM insulin was infused for 1600 sec. At the end, we depolarized the islets with 25 mM KCl for 240 sec to check their vitality. For the second protocol, after the equilibration, exogenous insulin of 0, 100, 500, or 1000 nM concentration was alternately infused for 120 sec or 150 sec with a period of 240 or 300 sec. The alternations were repeated 8 times, and at the end, the islets were depolarized with 25 mM KCl for 240 sec. To rule out the effect of physical stimuli during the solution exchange, we conducted control experiments in which we alternately exchanged the same glucose solution that contained exogenous insulin of 0 or 1000 nM concentration.

### Chemicals and reagents

Potassium chloride (KCl) and sodium chloride (NaCl) were purchased from Samchun Chemical (Seoul, South Korea). Calcium chloride (CaCl_2_), magnesium chloride (MgCl_2_), 4-(2-hydroxyethyl)-1-piperazineethanesulfonic acid (HEPES), and alpha-D-glucose were supplied by Sigma-Aldrich (Saint Louis, MO). Fetal bovine serum was obtained from Lonza (Walkersville, MD), and penicillin-streptomycin was purchased from Gibco (Carlsbad, CA). Fura-2 acetoxymethyl ester (fura-2/AM) was obtained from Invitrogen (F-1225; Eugene, OR). RPMI 1640 was purchased from Welgene (Seoul, South Korea). Collagenase P (from Clostridium histolyticum; 11213865001) was obtained from Roche Diagnostics (Indianapolis, IN). All of the solutions were prepared using Milli-Q deionized water (Millipore, 18.2 MΩ/cm at 25°C). All of the buffer solutions used in the experiments contained 125 mM NaCl, 5.9 mM KCl, 2.56 mM CaCl_2_, 1.2 mM MgCl_2_, 25 mM HEPES (pH 7.4), and various concentrations of glucose (3–15 mM as indicated). The buffer was supplemented with 1 mg/mL BSA (fraction V; USB 10857; Cleveland, OH). Bovine pancreas insulin (I6634) was purchased from Sigma-Aldrich (St. Louis, MO).

### Data analysis

We removed a linear trend in the time traces of the Ca^2+^ oscillations to focus on their phase by using the function “detrend” in MATLAB (MathWorks, Natick, MA). This modification set the mean of the time traces to zero. All the detrended Ca^2+^ profiles for different experimental protocols are available in Supporting Information (S1 File). To examine the effect of constant or alternating insulin stimuli, we analyzed the time traces between 1600 and 3600 sec, except for the constant infusion experiment of 100 nM insulin where we analyzed the time trace between 1600 and 3200 sec. We computed the duration of the continuous periods of positive values in the detrended time traces. The time fraction of the positive events was defined as the plateau fraction. We then applied the Fourier transformation to the detrended time traces and obtained their power spectra, and the maximum values defined their peak frequencies. We used the inverse value of the peak frequency as the dominant period. We used the time trace data only if their dominant periods were larger than 2 min and shorter than 12 min. To measure the degree of synchronization between islets, we calculated the Pearson correlation coefficient between the time traces of the islet Ca^2+^ oscillations. Note that the usual synchronization index based on the phase extraction with the Hilbert transform [34] did not work for analyzing the complex Ca^2+^ oscillations with fast spikes and slow oscillations at 8.3 mM glucose.

### Model simulation

We simulated the Ca^2+^ oscillation of islets by adopting the active rotator model,

$$\frac{d\theta }{dt}=\omega (I)-\mu (G)\cos\theta ,$$

where the phase *θ*(*t*) constantly increased with an intrinsic frequency *ω*(*I*), and the oscillation was modulated by a pinning force *μ*(*G*). In this study, we found that the intrinsic frequency was modulated by insulin, whereas the pinning force was modulated by glucose. First, in the absence of exogenous insulin (*I*_1_ = 0 nM), islets have heterogeneous intrinsic frequencies for their Ca^2+^ oscillations: *ω*(*I*_1_) follows a Gaussian distribution with a mean of 1.92 rad/min and standard deviation of 0.23 rad/min [10]. Then, exogenous insulin of concentration *I*_2_ = 1000 μM modulated the intrinsic velocity as *ω*(*I*_2_) = *νω*(*I*_1_), where we estimated the modulation factor *ν* = *ω*(*I*_2_)/*ω*(*I*_1_) = 4.6/5.8 ≈ 0.8 using the dominant periods, 2π/*ω*(*I*_1_) = 4.6 min and 2π/*ω*(*I*_2_) = 5.8 min for *I*_1_ = 0 and *I*_2_ = 1000 nM insulin infusions. Second, the pinning force was an increasing function of glucose, $\mu (G)=\bar{\mu }\sinh\left((G-G_{0}^{\mu })/\delta G^{\mu }\right)$, where $\bar{\mu }=0.10$ rad/min, $G_{0}^{\mu }=11.6$ mM and *δG*^*μ*^ = 1.2 mM [10]. Given a fixed *μ*(*G*_1_) = −0.78 rad/min for *G*_1_ = 8.3 mM, and *ω*(*I*_1_) = 1.92 rad/min and *ω*(*I*_2_) = 1.54 rad/min, we could estimate the plateau fractions,

$$\frac{2\int_{0}^{\pi /2}d\theta {\left[\omega (I)-\mu (G)\cos\theta \right]}^{-1}}{\int_{0}^{2\pi }d\theta {\left[\omega (I)-\mu (G)\cos\theta \right]}^{-1}},$$

0.37 for *I*_1_ = 0 nM and 0.33 for *I*_2_ = 1000 nM, which are close with the observed plateau fractions 0.39±0.03 and 0.36±0.04, respectively. We simulated one hundred independent rotators following the above equation with random initial phases θ(0) ∈ [0,2π]. The initial glucose and insulin were set to *G*_1_ = 8.3 mM and *I*_1_ = 0 nM, and the rotators were equilibrated for 10 min. Afterward, we stimulated the rotators with a high glucose (*G*_2_ = 8.8 mM) and/or high insulin (*I*_2_ = 1000 nM) for 2 min with a 4-min period. We repeated the same simulation with a lower concentration (*I*_2_ = 100 nM) of insulin stimulus with a modified modulation factor ν = ω(*I*_2_)/ω(*I*_1_) = 4.6/5.3 ≈ 0.87 using the dominant periods, 2π/ω(*I*_1_) = 4.6 min and 2π/ω(*I*_2_) = 5.3 min for *I*_1_ = 0 and *I*_2_ = 100 nM insulin infusions. The differential equations were numerically integrated using the 2^nd^ order Runge-Kutta method with a sufficiently small time step, Δ*t* = 0.01 [35].

### Statistical analysis

Differences between unpaired observations were evaluated with Student’s *t*-test. The data in the text are presented as the means ± standard deviations. Findings were considered statistically significant at P < 0.01.

## Supporting information

S1 File Detrended Ca^2+^ trace data. All the time traces of detrended Ca^2+^ oscillations for constant insulin infusion (0, 100, or 1000 nM) and alternating insulin infusion (100, 500, or 1000 nM with 4 or 5 min periods) were compressed as a single file, S1_File.zip, in which each imaging experiment was recorded as a text file where the first column represents time (sec) and the following columns represent detrended Ca^2+^ traces from multiple islets.
(ZIP)
